# Supplementary material for: What’s the relative humidity in tropical caves?
Source: PLoS One. 2021 Sep 22;16(9):e0250396. doi: 10.1371/journal.pone.0250396 (PMC8457490; doi:10.1371/journal.pone.0250396)
Supplement: Attachment — Submitted filename: Response to Reviewers-2.docx [file pone.0250396.s003.docx]

With respect to the Reviewer’s comments (please note the line numbering seems a bit haywire in the track changes version with some gaps in numbering between pages):

1. We have added discussion of climate change at the end of the discussion as well as in the introduction. We mention the linkage of temperature and RH via the Clapeyron equation in line 102.

2. With respect to Badino’s work on supersaturated atmospheres in caves (clouds in caves), we discuss this in connection with condensation corrosion (line 595), and mention it elsewhere in the manuscript.

3. The description of GLMM (line 265) was rewritten and expanded, and ar(1) is explained.

4. The acronyms in Table 1 are defined at the bottom of the Table.

5. The relationship between RH and fauna (or more correctly, the lack thereof) is discussion beginning on line 438.

6. We include the possibility that the winter effect is minimal because of reduced external seasonal variation in tropical climate on line 519.

7. We include a mention of the hurricane effect on line 555

8. We have added five additional recent references on global warming in caves.

Please let me know if more information is required.

Sincerely,

David C Culver
