# Supplementary material for: What’s the relative humidity in tropical caves?
Source: PLoS One. 2021 Sep 22;16(9):e0250396. doi: 10.1371/journal.pone.0250396 (PMC8457490; doi:10.1371/journal.pone.0250396)
Supplement: Attachment — Submitted filename: Response to Reviewers 2.docx [file pone.0250396.s004.docx]

Reviewer #1 had no additional comments.
